# Metabolomics Distinguishes DOCK8 Deficiency from Atopic Dermatitis: Towards a Biomarker Discovery

**DOI:** 10.3390/metabo9110274

**Published:** 2019-11-12

**Authors:** Minnie Jacob, Xinyun Gu, Xian Luo, Hamoud Al-Mousa, Rand Arnaout, Bandar Al-Saud, Andreas L. Lopata, Liang Li, Majed Dasouki, Anas M. Abdel Rahman

**Affiliations:** 1 Department of Genetics, King Faisal Specialist Hospital and Research Center (KFSH-RC), Riyadh 11211, Saudi Arabia; minnie@kfshrc.edu.sa; 2 Australian Institute of Tropical Health and Medicine, James Cook University, Townsville QLD 4814, Australia; andreas.lopata@jcu.edu.au; 3 Department of Chemistry, University of Alberta, Edmonton, AB T6G 2R3, Canadaxluo2@ualberta.ca (X.L.); rarnaout@kfshrc.edu.sa (R.A.); liang.li@ualberta.ca (L.L.); 4 Section of Pediatric Allergy and Immunology, Department of Pediatrics, King Faisal Specialist Hospital & Research Centre (KFSH-RC), Riyadh 11211, Saudi Arabia; hamoudalmousa@kfshrc.edu.sa (H.A.-M.); balsaud@kfshrc.edu.sa (B.A.-S.); 5 College of Medicine, Alfaisal University, Riyadh 11533, Saudi Arabia; 6 Department of Chemistry, Memorial University of Newfoundland, St. John’s, NL A1B 3X7, Canada

**Keywords:** 3-hydroxyanthranilic acid, dansylation, dedicator of cytokinesis, hypotaurine, liquid chromatography-mass spectrometry, metabolomics

## Abstract

Bi-allelic mutations in the dedicator of cytokinesis 8 (*DOCK8*) are responsible for a rare autosomal recessive primary combined immunodeficiency syndrome, characterized by atopic dermatitis, elevated serum Immunoglobulin E (IgE) levels, recurrent severe cutaneous viral infections, autoimmunity, and predisposition to malignancy. The molecular link between DOCK8 deficiency and atopic skin inflammation remains unknown. Severe atopic dermatitis (AD) and DOCK8 deficiency share some clinical symptoms, including eczema, eosinophilia, and increased serum IgE levels. Increased serum IgE levels are characteristic of, but not specific to allergic diseases. Herein, we aimed to study the metabolomic profiles of DOCK8-deficient and AD patients for potential disease-specific biomarkers using chemical isotope labeling liquid chromatography-mass spectrometry (CIL LC-MS). Serum samples were collected from DOCK8-deficient (*n* = 10) and AD (*n* = 9) patients. Metabolomics profiling using CIL LC-MS was performed on patient samples and compared to unrelated healthy controls (*n* = 33). Seven metabolites were positively identified, distinguishing DOCK8-deficient from AD patients. Aspartic acid and 3-hydroxyanthranillic acid (3HAA, a tryptophan degradation pathway intermediate) were up-regulated in DOCK8 deficiency, whereas hypotaurine, leucyl-phenylalanine, glycyl-phenylalanine, and guanosine were down-regulated. Hypotaurine, 3-hydroxyanthranillic acid, and glycyl-phenylalanine were identified as potential biomarkers specific to DOCK8 deficiency. Aspartate availability has been recently implicated as a limiting metabolite for tumour growth and 3HAA; furthermore, other tryptophan metabolism pathway-related molecules have been considered as potential novel targets for cancer therapy. Taken together, perturbations in tryptophan degradation and increased availability of aspartate suggest a link of DOCK8 deficiency to oncogenesis. Additionally, perturbations in taurine and dipeptides metabolism suggest altered antixidation and cell signaling states in DOCK8 deficiency. Further studies examining the mechanisms underlying these observations are necessary.

## 1. Introduction

Dedicator of cytokinesis 8 (DOCK8) deficiency is caused by the loss of function mutations in the dedicator of cytokinesis 8 (*DOCK8*) gene [1,2], characterized by susceptibility to sinopulmonary infections, atopic eczema, asthma, food allergies, severe viral infections, and increased incidence of malignancy leading to premature death [2,3]. DOCK8 is a cytoskeletal protein, which contains two related conserved protein domains DHR1 and DHR2, with bi-allelic *DOCK8* mutations having both been reported with frequent large deletions and point mutations, leading to protein loss of function [2,4]. It is highly expressed in the immune system, especially in lymphocytes, but is also expressed in the placenta, kidney, lung, and pancreas [5]. DOCK8-deficient patients develop atopic dermatitis, asthma, and severe allergies to food and environmental antigens in early infancy [6]. Chronic viral infections are also distinctive features with the common pathogens being herpes simplex virus (HSV), human papillomavirus (HPV), molluscum contagiosum virus (MCV), and varicella-zoster virus (VZV). In general, all DOCK8-deficient patients are susceptible to recurrent sinopulmonary infections caused by a wide variety of pathogens including Streptococcus pneumoniae, Haemophilus influenzae, Pneumocystis jirovecii, Histoplasma capsulatum, and Legionella pneumophila [7].

Atopic dermatitis (AD) or eczema is a prevalent pediatric chronic inflammatory skin disease and specific food allergens and nutrients are closely related to the development and severity of this disease. AD is characterized by intense pruritus, occurring primarily in infants and children, with approximately 70% of cases starting before the age of five years. Eczema classically involves the face, scalp, and extensor surfaces of extremities. Impaired innate and adaptive immunity, environmental changes, and alterations in genes involved in epidermal barrier functions contribute towards the clinical manifestations of this disease [8]. These patients are susceptible to superficial infections with *Staphylococcus aureus*, but invasive infections rarely occur in AD, unlike DOCK8-deficient patients. Treatment of AD is directed mainly towards the prevention and management of infection and immunomodulation to control the associated rash and pruritus. Topical corticosteroids, systemic antibiotics, and antifungal agents are used for both prophylactic and symptomatic treatment in conjunction with topical therapy. Atopic dermatitis and DOCK8 deficiencies share similar clinical symptoms including eczema, eosinophilia, and characteristic elevated levels of serum Immunoglobulin E (IgE).

Metabolomics is a rapidly growing and promising discipline enabling the quantification of the group of small molecules involved in intermediary metabolism encoded by genomic DNA. Over the last decade, both targeted and untargeted metabolomics studies have identified several relevant biomarkers involved in complex clinical phenotypes in diverse biological systems. Significant environmental and clinical disturbances can be monitored at the metabolomic level by examining an array of different pathways that are crucial for cellular homeostasis [9,10]. As the metabolome is complex and dynamic, newer and more reliable quantitative technologies have enabled the discovery of biomarkers specific enough to distinguish patients in various health states from healthy subjects [11,12,13]. 

Chemical isotope labeling liquid chromatography-mass spectrometry (CIL LC-MS) is a robust and emerging analytical platform used in biomarker discovery, where different labeling reagents are used to target functional groups based on sub-metabolomes [14,15].

Apart from cytokine biomarkers capable of distinguishing DOCK8-deficient from AD patients [16], definitive metabolomics biomarkers have not been identified yet. Therefore, we aimed to employ in-depth metabolomics technologies to study the metabolomic profiles of a cohort of patients with DOCK8 deficiency and severe AD to explore biomarkers that potentially reflect disease pathogenesis and may contribute towards improved disease monitoring, and ultimately novel clinical interventions. We, therefore, applied CIL LC-MS targeting the amine/phenol sub-metabolomes to identify novel and differentially expressed biomarkers in hereditary DOCK8-deficient and AD patient groups.

## 2. Results

### 2.1. Clinical Characterizations in DOCK8-Deficient and AD Patients

The clinical and laboratory characteristics of the study cohorts are represented in Table S1. The mean age of the DOCK8-deficient and AD cohort was 13.2 ± 5.9 and 10.8 ± 1.4 years, respectively. Whereas the mean age of healthy controls collected from adults was 23 ± 1.03 (Table S1). Comparatively, the CD4+/CD8+ white blood cells count ratio in the DOCK8-deficient cohort (2.8 ± 0.99) was higher compared with that of the AD cohort (1.43 ± 0.14). While eosinophilia was present in all patients, the counts were significantly different in the DOCK8-deficient cohort compared with AD. On the other hand, there is no significant difference in neutrophil counts between the two cohorts. The mean red blood cells (RBC) and white blood cellsWBC counts in DOCK8-deficient patients were 4.5 ± 0.5 (10^12^/L) and 10.53 ± 2.3 (10^9^/L), respectively, whereas in AD patients, they were 5.3 ± 0.16 (10^12^/L) and 6.74 ± 0.9 (10^9^/L), respectively. The Severity Scoring of Atopic Dermatitis (SCORAD) and the Visual Analogue Scale (VAS) pruritus scores were calculated for both DOCK8-deficient and AD groups (Table S1) (table was reproduced with permission) [16].

**Figure 1:**
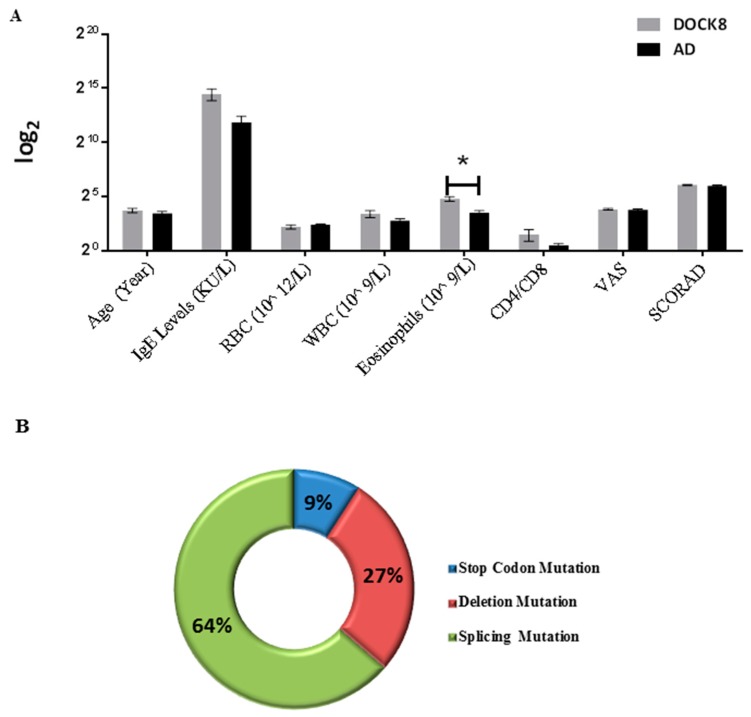
Demographic profiles in dedicator of cytokinesis 8 (DOCK8)-deficient and atopic dermatitis (AD) patients: (**A**) Only blood eosinophil counts were statistically different between DOCK8-deficient and AD patients. (**B**) Distribution of mutations in DOCK8-deficient patients. (one-way analysis of variance (ANOVA), post hoc Tukey’s method, ** *p*-value < 0.05, * *p*-value < 0.001). SCORAD, Severity Scoring of Atopic Dermatitis; VAS, Visual Analogue Scale. (measuring itch intensity).

The most commonly seen clinical presentations in our DOCK8-deficient cohort were atopic dermatitis, food allergies, pneumonia, and staphylococcal infections, whereas in AD patients, pneumonia or invasive staphylococcus infections were not observed. As anticipated, total IgE levels in both DOCK8-deficient and AD groups were elevated when compared with the control group, with DOCK8-deficient patients showing significantly higher serum IgE levels (*p*-value < 0.05) compared with AD patients and controls (5–500 KU/L), and eosinophil levels were seen to be significantly up-regulated in DOCK8-deficient patients compared with AD (Figure 1A). Among the DOCK8-deficient cohort, splicing mutations were the most common (64%), followed by deletion mutations (27%) and stop codon mutations (9%) (Figure 1B).

### 2.2. Metabolomics Profiling

**Figure 2:**
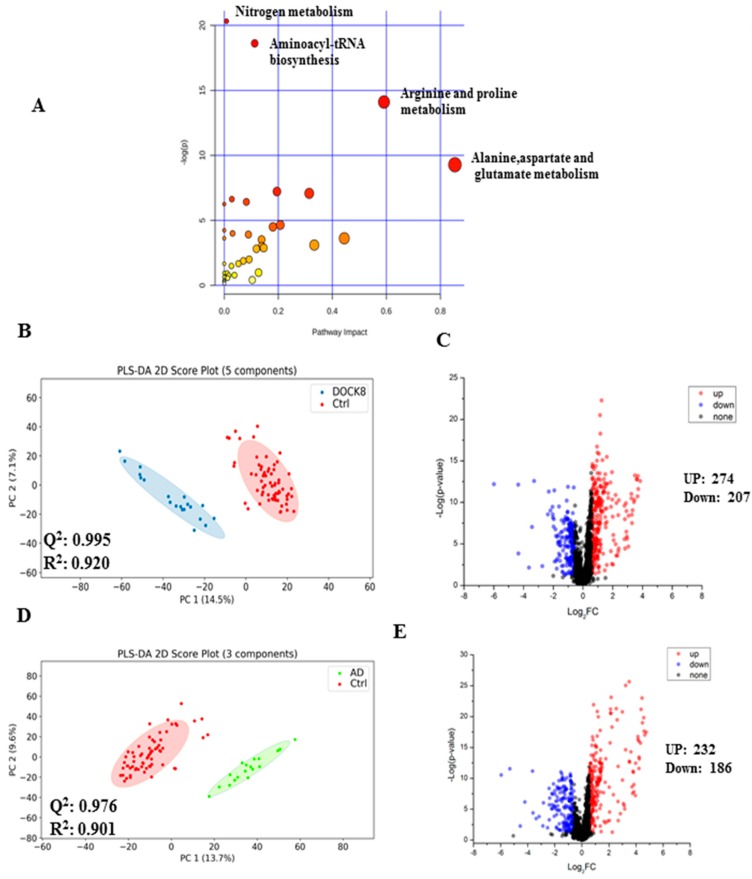
Pathway analysis and binary comparisons in serum of patients with DOCK8 deficiency and atopic dermatitis. (**A**) Pathway analysis for DOCK8-deficient patients vs. Ctrl comparison. (**B**) DOCK8-deficient (*n* = 10 run in duplicates) vs. Control (*n* = 33 run in duplicates). PLS-DA score plot with a calculated space R^2^ = 0.995 and Q^2^ = 0.920. Five latent variables were used because it has the highest cross-validation Q^2^ value. (**C**) Volcano plots (DOCK8 deficient patients vs. control) with fold change >1.5 (up-regulated = 274 metabolites) and <0.67 (down-regulated = 207 metabolites); *q* = 0.049, *p* = 0.107, 40 metabolites were positively identified. (**D**) Atopic dermatitis (AD) (*n* = 9) vs. Control (*n* = 33). PLS-DA score plot, with a calculated space R^2^ = 0.976 and Q^2^ = 0.901, three latent variables were used because it has the highest cross-validation Q^2^ value. (**E**) Volcano plot with fold change >1.5 (up-regulated = 232) and <0.67 (down-regulated = 186), *q* = 0.050, *p* = 0.055, a total of 37 metabolites were positively identified. Abbreviations: DOCK8, dedicator of cytokinesis 8; AD, atopic dermatitis; PLS-DA, partial least square discrimination analysis; PC, principal component.

Pathway analysis (Figure 2A) identified nitrogen (global) amino acid metabolism pathways to be the most perturbed, followed by an amino acyl-tRNA biosynthesis when DOCK8 deficiency was compared with AD and Ctrl. The global metabolomics profile was dissected in several binary analyses for a better understanding of the distinctive contribution of each gene in the DOCK8-deficient group compared with either the AD or Ctrl groups. The partial least square-discriminant analysis (PLS-DA) score plot demonstrates significant separation between the DOCK8-deficient and Ctrl groups (Figure 2B). The univariate and volcano plot analyses were also performed and a total of 3438 metabolites features were detected; among them, a group of metabolites (*n* = 481) was differentially expressed and visualized in the volcano plot (Figure 2C). The cutoff *p*-value has a corresponding q-value of less than 0.05 and a fold change cutoff value of 1.5. Among the 481 dysregulated metabolites, 274 metabolites were up-regulated, while 207 metabolites were down-regulated in the DOCK8-deficient group (Figure 2C). However, only 40 metabolites were positively identified using the dansyl standard library based on the exact mass and retention time match for the metabolite and its labeled internal standard (Table S2).

Similarly, the binary comparison between AD patients and Ctrl groups (Figure 2D) showed clear cluster separation between the two groups (Q^2^ = 0.976), and a total of 418 metabolites were dysregulated, including 232 up-regulated and 186 down-regulated metabolites (Figure 2E).

**Figure 3:**
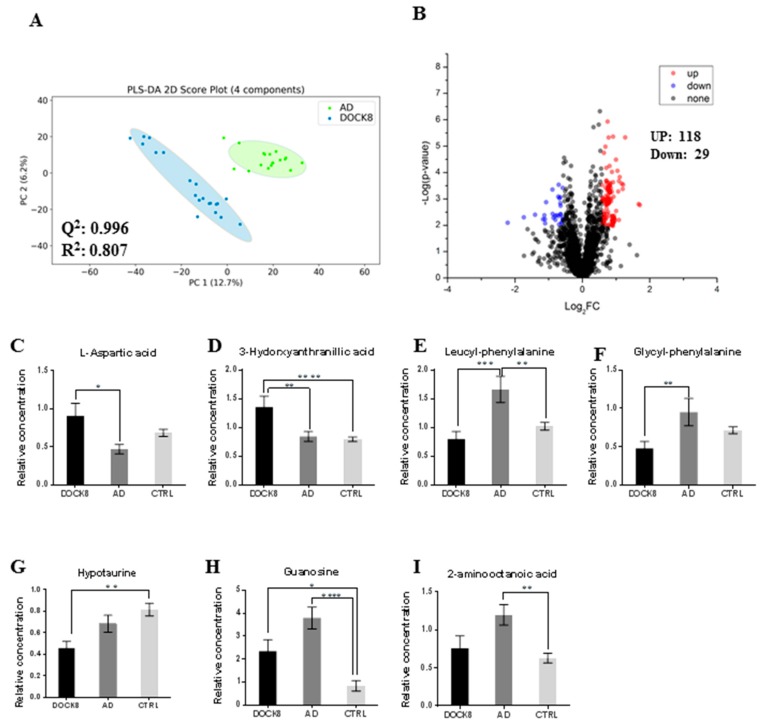
Positively identified serum metabolites in DOCK8-deficient patients vs. AD vs. Ctrls. (**A**) PLS-DA score plot for binary comparison between DOCK8-deficient patients and AD, with a calculated space Q^2^ = 0.996 and R^2^ = 0.807, four latent variables were used as they have the highest cross-validation Q^2^ value. (**B**) Volcano plot analysis with fold change >1.5 (up-regulated = 118) and <0.67 (down-regulated = 29), a total of seven metabolites were positively identified. (**C**) L-Aspartic acid is up-regulated in DOCK8-deficient patients compared with AD patients. (**D**) 3-Hydroxyxanthranillic acid is up regulated in DOCK8-deficient patients. Dipeptides leucyl-phenylalanine and glycyl-phenylalanine are up-regulated in AD patients compared with DOCK8-deficient (**E**, **F**, respectively). (**G**) Hypotaurine is down-regulated in DOCK8-deficient patients compared with Ctrl. (**H**) Guanosine is up-regulated in DOCK8-deficient and AD patients, while 2-aminooctanoic acid is up-regulated in AD patients only. (**I**) For paired analysis, a combination of t-test and fold change analyses are represented in this volcano plot, where the *x*-axis, False discovery rate (FDR-corrected *p*-value) and the *y*-axis are true positive. Statistical analysis was performed using one-way ANOVA and post hoc Tukey’s test, where * indicates significance with *p*-value < 0.05, ** *p*-value < 0.001, and otherwise not significant (ns). Abbreviations: DOCK8, dedicator of cytokines 8; AD, atopic dermatitis; Ctrl, healthy controls; PLS-DA, partial least square discrimination analysis.

In this group, only 37 metabolites were positively identified using the dansyl standard library (Table S3). Seven metabolites were positively identified using the dansyl standard library after a binary comparison between DOCK8-deficient and AD cohorts (Figure 3A,B), while a total of 147 metabolites were dysregulated (118 and 29 metabolites were up and down-regulated in DOCK8 deficiency group, respectively). The seven positively identified metabolites are presented in Figure 3C–I (Table S4). Among those, aspartic acid and 3-hydroxyanthranillic acid were significantly up-regulated in DOCK8-deficient patients, whereas the dipeptides leucyl-phenylalanine and glycyl-phenylalanine were down-regulated compared with the AD patients. Hypotaurine, guanosine, and 2-aminooctanoic acid were not found to be significantly differentially expressed in DOCK8 deficiency compared with AD after using one-way analysis of variance (ANOVA)/post-Tukey’s method (Figure 3G–I).

### 2.3. Biomarker Evaluation

**Figure 4:**
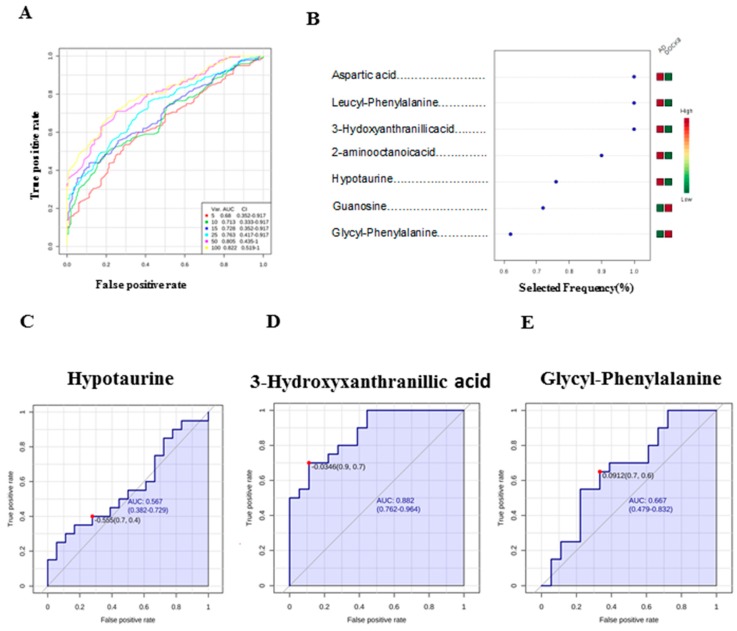
Receiver operating characteristics (ROC) curve and loading plots for positively identified serum metabolites in the comparison between DOCK8-deficient and AD patients. (**A**) ROC was generated by the PLS-DA model showing an area under the curve (AUC) = 0.822. (**B**) Frequency plot with seven positively identified metabolites. (**C**) Hypotaurine is not significantly expressed in DOCK8-deficient patients, AUC = 0.567 and *p*-value of 0.41537. (**D**) 3-Hydroxyxanthranillic acid is up-regulated in DOCK8-deficient patients, AUC = 0.882 and *p*-value of 4.4491 × 10^−5^. (**E**) Glycyl-phenylalanine is down-regulated in DOCK8-deficient patients compared with AD patients, AUC = 0.667 and *p*-value of 0.04766. Data were normalized; transformed; and scaled by median, log, and Pareto scaling to make sure all the data are under the Gaussian distribution. For paired analysis, a combination of t-test and fold change analyses are represented, where the *x*-axis (FDR-corrected *p*-value) and the *y*-axis are true positive. **Abbreviations:** DOCK8, dedicator of cytokines8; AD, atopic dermatitis; Ctrl, healthy controls.

As a result of the binary comparisons between DOCK8-deficient versus Ctrl, AD versus Ctrl, and DOCK8-deficient versus AD groups, receiver operating characteristics (ROC) exploring curves were generated (Figure 4A). Multivariate exploratory ROC analysis was generated using PLS-DA as a classification and feature ranking methods. The combination of the top metabolites in ROC curves shows areas under the curve (AUCs) ranging from 0.68–0.82 (Figure 4A). The significant features of the positively identified metabolites (Figure 4B) show aspartic acid and 3-hydroxyanthranilic acid to be up-regulated, whereas hypotaurine, leucyl-phenylalanine, glycyl-phenylalanine, guanosine, and 2-aminooctanoic acids were found to be down-regulated in DOCK8 deficiency. Hypotaurine is not significant (Figure 4C), whereas 3-hydroxyanthranillic acid is up-regulated (Figure 4D) compared with glycyl-phenylalanine (Figure 4E), and is down-regulated in DOCK8-deficient patients. The combination of all seven analytes gave the maximum confidence of differentiation and detection of DOCK8-deficient from the AD with an AUC = 0.922.

Previous studies have provided evidence demonstrating the influence of age and sex on the metabolome, both cross sectionally and longitudinally [17,18,19,20,21,22]. Therefore, it is very important to match the healthy control and affected subjects for age and sex, among other variables, to avoid such an effect. In this study, most of the children who were eligible for sample collection were not as healthy as they should be “based on the study design”. Therefore, we used the youngest healthy adults we could recruit as controls. When we compared each of the patients’ groups with Ctrl, we only showed the differences without explicit interpretation owing to the age and gender confounding factors. However, the age difference between the study groups is not too huge compared with the differences between the 40 metabolites (DOCK8 vs. Ctrl) or the 37 metabolites (AD vs. Ctrl) (data not shown). When we compare the two groups of the dysregulated metabolites for the two comparisons (the lists of 40 and 37 metabolites), we concluded that age is not an effector on the reported metabolites. However, the gender might still have an effect on some metabolites, which were excluded from our final list of biomarkers.

## 3. Discussion

It is critical to recognize DOCK8 deficiency and differentiate its various clinical and molecular forms before severe life-threatening complications arise. For example, Aydin, et al. (2015), in a study on 136 DOCK8-deficient patients, reported malignancies in 17%, life-threatening infections in 58%, and non-infections cerebral events in 10% of their patients [23]. Differentiating DOCK8-deficient from AD patients can be difficult in infants and young children because of overlapping clinical and laboratory findings. The DOCK8 protein regulates intracellular signaling networks, proliferation, differentiation, migration, synapsis formation, adhesion, and survival of affecting innate and adaptive immunity reflecting complex function [3,24,25].

The identification of predictive biomarkers to distinguish DOCK8-deficient from AD, based on serum metabolite changes, requires a highly sensitive platform to allow the detection of very low abundant (pmol to fmol) metabolites. Chemical isotope labeling LC-MS represents a robust method for metabolomics profiling and biomarker discovery, as the 13C-labeled pool served as an internal standard and compensated for the fluctuations in MS response [26]. In this study, seven metabolic features were found to significantly differentiate between DOCK8-deficient and AD patients. Taken together, these seven differentially expressed metabolites paint a distinctive metabolomics profile in DOCK8-deficient and AD patients (Figure 3C–I). Up-regulation of 3-hydroxyanthranilic acid was observed in the DOCK8-deficient cohort compared with Ctrl and AD, while aspartic acid was up-regulated in DOCK8-deficient cohort compared with Ctrl and hypotaurine was down-regulated in DOCK8 deficiency compared with the AD.

The binary analyses between DOCK8 deficiency with and without various clinical complications (asthma, bronchiectasis, molluscum contagiosum, sclerosing cholangitis, candidiasis, warts, sinusitis, or malignancy) failed to demonstrate a secondary role for these phenotypes on the overall DOCK8 deficiency-specific metabolites (Figure S1), which suggests that these metabolites are primarily the result of the underlying genetic deficiency, rather than occurring as a result of secondary medical complications.

The hypotaurine–taurine metabolism pathway starts with cystamine production via cystine decarboxylation, which is then reduced to cysteamine followed by oxidation to hypotaurine (by the enzyme cysteamine dioxygenase). Hypotaurine is finally oxidized to the final product taurine by hypotaurine dehydrogenase, which is then excreted out of the body or used within. While very little is known about the physiological role of hypotaurine, taurine is known to have diverse cellular functions including neurotransmission, retinal photoreceptor differentiation (through the taurine upregulated gene 1 (TUG1), a non-coding RNA that modulates the expression of photoreceptor-specific genes in the retina [27], osmoregulation, calcium modulation, and suppression of inflammation, as well as normal mitochondrial respiratory chain function [28]. Within the central nervous system CNS, taurine exhibits an age-dependent gradient expression and has its own synthesizing enzymes, receptors, and transporters [29]. Taurine has also been implicated as a tumor marker in many different cancer types [30,31,32,33]. The exact mechanisms regulating taurine levels in tumors have not been established, but may involve either regulation of its synthesis from hypotaurine and/or regulation of its uptake from the extracellular environment, mediated by the taurine transporter SLC6A6. Holopainen et al. (1982) demonstrated the rapid uptake of hypotaurine into neuroblastoma cells, suggesting that hypotaurine may have a function in the regulation of neuronal activity [34]. Other studies suggested a role for hypotaurine as an antioxidant and protective agent under physiological conditions [35,36]. Peng et al. (2016) also showed that under hypoxic signaling, hypotaurine behaves as an oncometabolite promoting tumor progression [37]. The observation of under expression of hypotaurine in the DOCK8-deficient patients suggests a potential loss of its antioxidant and protective effects.

3-Hydroxyanthranillic acid (3-HAA), a tryptophan catabolism molecule produced through the kynurenine pathway, suppresses antitumor immunity in human malignancy [38], has immune regulatory properties as it can inhibit Th1 and Th2 cells, increases the percentage of regulatory T-cells, and regulates leukocyte infiltration and plaque formation [39]. It is found in the human epidermis, where it participates in multiple enzymatic reactions [40,41]. Also, 3-HAA appears to play an essential role in the pathogenesis of several inflammatory, infectious, and degenerative diseases [42]. The increased tryptophan catabolism, concerning infections during the disease, may lead to increased levels of 3-HAA, as seen in our DOCK8-deficient patients (Figure 4D).

Perturbations in amino acid metabolism had also been observed in some cancers as well as neurodegenerative disorders such as Alzheimer’s disease and Parkinson’s diseases [43,44,45]. Aspartic acid is a major excitatory neurotransmitter, which was increased in some epileptic and stroke patients, and decreased in patients with depression and brain atrophy. In contrast, guanosine is a nucleoside that exerts important neuroprotective and neuromodulatory roles in the central nervous system, which may be related to inhibition of the glutamatergic neurotransmission activity. Glycyl-phenylalanine, a dipeptide produced by incomplete protein catabolism, consists of glycine and phenylalanine and is known to play an essential role in cell signaling effects by influencing specific amino acid degradation pathways [46]. It is transported intact by a cation-independent facilitative diffusion mechanism, during which the dipeptide is hydrolyzed to its component amino acids [46]. Some dipeptides have physiological or cell-signaling effects, although most are short-lived intermediates on their way to specific amino acid degradation pathways following further proteolysis. This dipeptide has not yet been identified in human tissues or biofluids, and so it is classified as an ‘expected’ metabolite.

2-aminooactanoic acid is shown to be perturbed in human colorectal cancers [47]. The differential expression of aspartate and 3-hydroxyanthranilic acids observed in our DOCK8-deficient patients is supported by recently reported data [48,49]. Taken together, these findings call for further analysis of the perturbed amino acid pathways for additional insight into its significance.

## 4. Material and Methods

### 4.1. Chemicals

The LC-MS grade reagents, including water, acetonitrile (ACN), methanol, and formic acid, were purchased from Fisher Scientific (Ottawa, Canada) and 13C dansyl-chloride was available from the University of Alberta (http://mcid.chem.ualberta.ca).

### 4.2. Characteristics of the Study Population

Through the allergy/immunology clinics at King Faisal Specialist Hospital and Research Center (KFSHRC), children and adults with a genetically confirmed diagnosis of hereditary DOCK8-deficient and AD patients meeting the Hanifin and Rajka clinical criteria [50]. Healthy controls (adults) who visited the clinic for routine clinical care and those who were free of eczema, asthma, allergies, and infections were consented and recruited to participate in this study. Patients who received bone marrow transplantation, were enrolled in another clinical study, were unwilling to provide informed consent, or whose sample amount was not sufficient were excluded from the study. A baseline questionnaire including clinical symptoms, allergies, and family history was collected. This study was approved by the Research Ethics Committee, at the Office of Research Affairs of King Faisal Specialist Hospital and Research Center. (KFSH&RC) (RAC No. 2160 015).

### 4.3. LC-MS

**Figure 5:**
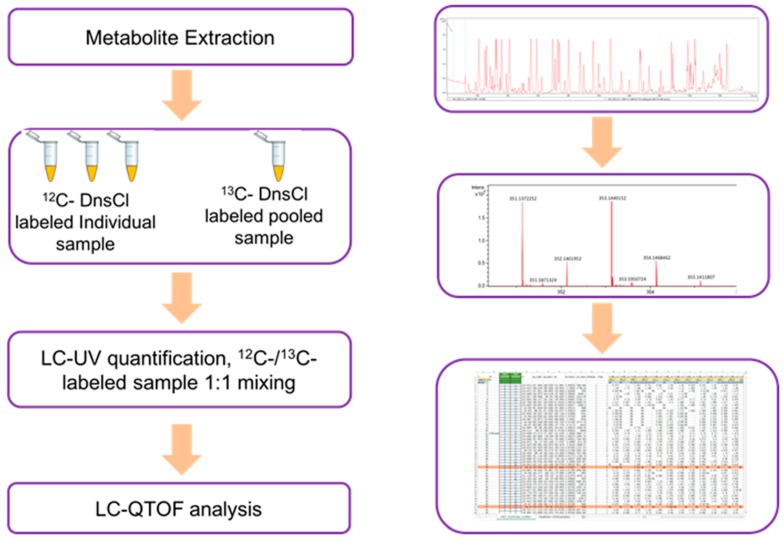
Flowchart of the metabolomics workflow. LC, liquid chromatography; QTOF, quadrupole time-of-flight.

In this CIL LC-MS metabolomics workflow (Figure 5), each sample was labeled by 12C dansyl-chloride (DnsCl), while a pooled sample was generated by mixing all individual samples, and then labelling by 13C DnsCl [14]. The 13C-labeled pooled sample served as a reference for all the 12C-labeled individual samples. Each sample was normalized before LC-MS analysis. LC-UV quantitation was performed to determine the total concentration of dansyl-labeled metabolites. Each 12C-labeled sample was mixed with the same molar amount of 13C-labeled pooled sample and injected into LC-MS. All labeled metabolites were identified as peak pairs on mass spectra, and the peak area ratios were used for quantitative metabolomic analysis.

The serum samples were analyzed using a Thermo Fisher Scientific Dionex Ultimate 3000 UHPLC System (Sunnyvale, CA, USA) linked to a Bruker Maxis II quadrupole, time-of-flight (Q-TOF) mass spectrometer (Bruker, Billerica, UK). The LC column was an Agilent reversed-phase Eclipse plus C18 column (2.1 mm × 10 cm, 1.8 μm particle size, 95 Å pore size), while the mobile phase A was 0.1% (*v/v*) formic acid in 5% (*v/v*) ACN, and solvent B was 0.1% (*v/v*) formic acid in acetonitrile. The LC gradient was as follows: t = 0 min, 20% B; t = 3.5 min, 35% B; t = 18 min, 65% B; t = 21 min, 99% B; t = 34 min, 99% B, with a flow rate of 0.18 mL/min. The MS conditions were as follows: polarity, positive; dry temperature, 230 °C; dry gas, 8 L/min; capillary voltage, 4500 V; nebulizer, 1.0 bar; endplate offset, 500V; spectra rate, 1.0 Hz.

### 4.4. Data Collection, Processing, and Analysis

The LC-MS spectra were first converted to CSV files by Bruker Daltonics Data Analysis 4.3 Software, UK and peak pairs were extracted from CSV files by IsoMS. Meanwhile, the redundant pairs (e.g., those of Na^+^, NH3^+^ adduct ions, and dimers) were filtered out [51]. All data generated from multiple runs were aligned together based on the peak’s accurate mass and retention time.

The missing values in the aligned file were filled by Zerofill software [52]. A univariate analysis (volcano plot) was performed for each binary comparison to identify significantly differentially expressed metabolites. Here, we used a criterion of fold-change of greater than 1.5 or less than 0.67 with q-value (false discovery rate) less than 0.05. The q-value is calculated by R script based on *p*-value from a *t*-test. In the volcano plot, the *x*-axis represents the fold change (FC) between two comparison groups, and the *y*-axis represents the *p*-value. The principal component analysis (PCA) and partial least squares discriminant analysis (PLS-DA) plots were performed using Iso MS Pro. (NovaMT Inc.) The metabolites were positively identified by searching against DnsID Library (www.mycompoundid.org) using retention time and accurate mass [53]. Putative identification was performed by searching accurate mass against MyCompoundID library, which contains 8021 known human metabolites and 375,809 predicted metabolites (www.mycompoundid.org) [54]. Analysis of variance (ANOVA) using post-hoc Tukey’s method of analysis, with multiplicity-adjusted *p*-values, for each comparison, was executed for the statistical analysis among the three groups. This method was chosen not only because of the unequal group sizes among the experimental and the control groups, but also because it reduces the probability of making a type 1 error and supports testing of pairwise differences. Further analysis was performed on GraphPad Prism (version 6.0, Graph Pad software, LA Jolla, CA).

The receiver operating characteristic (ROC) curves were constructed using random forest method MetaboAnalyst software version 3.0 (McGill University, Montreal, Canada) (http://www.metaboanalyst.ca) for global analysis. The raw data were normalized, transformed, and scaled by a median, log, and Pareto, respectively, to make sure all the data are visualized under Gaussian distribution.

## 5. Conclusions

DOCK8 deficiency appears to be associated with a distinctive metabolomics profile characterized by significant differential overexpression of 3-HAA and aspartic acid, both of which have been linked to oncogenesis coupled with underexpression of hypotaurine, guanosine, and the dipeptides leucyl-phenylalanine and glycyl-phenylalanine, which together seem to contribute to some of the immune and malignancy-related phenotypes observed in this disease. The complex nature of these diseases suggests that no single biomarker will be sufficient to meet the clinical needs of such patients; instead, a larger panel of biomarkers will ultimately be required. These findings may inform further mechanistic analyses in these diseases.

## Supplementary Materials

The following are available online at https://www.mdpi.com/2218-1989/9/11/274/s1, **Table S1:** Summary of clinical scores (VAS and SCORAD) and laboratory findings in DOCK8 deficient and atopic dermatitis cohorts, **Table S2:** Positively identified metabolites that have been significantly changed between the DOCK8 deficient patients and healthy controls. q-value < 0.05, **Table S3:** Positively identified metabolites that have been significantly changed between AD and Control, q-value < 0.05, **Table S4:** identified metabolites that have been significantly changed between the DOCK8 deficient and AD patients. q-value < 0.05, **Figure S1A:** PLS-DA loading plots based on binary comparisons in DOCK8-deficient patients with (**+**) and without (**-**) various clinical phenotypes including (**A**) Asthma, (**B**) Bronchiectasis, (**C**) Molluscum contagiosum, (**D**) Sclerosing cholangitis, **Figure S1B:** PLS-DA loading plots based on binary comparisons in DOCK8-deficient patients with (**+**) and without (**-**) various clinical phenotypes including (**E**) Candidiasis, (**F**) Warts, (**G**) Sinusitis, (**H**) Malignancy.
