# Supplementary material for: Metabolomics Distinguishes DOCK8 Deficiency from Atopic Dermatitis: Towards a Biomarker Discovery
Source: Metabolites. 2019 Nov 12;9(11):274. doi: 10.3390/metabo9110274 (PMC6918408; doi:10.3390/metabo9110274)
Supplement: Supplementary file 1 [file metabolites-09-00274-s001.pdf]

**Table S1.** Summary of clinical scores (VAS and SCORAD) and laboratory findings in DOCK8 deficient and atopic dermatitis cohorts.

Abbreviations: AD atopic dermatitis, DOCK8 dedicator of cytokinesis, F female, M male, SCORAD (severity scoring of atopic dermatitis), SEM standard error of the mean, VAS (visual analogue score).

| Diagnosis | Patient code | Mutation | Age (Y) | Sex (M/F) | IgE Levels (KU/L) | RBC 10^ 12/L | WBC 10^9/L | Eosinophils 10^9/L | CD4/CD8 Ratio | VAS | SCORAD |
|---|---|---|---|---|---|---|---|---|---|---|---|
| DOCK8 | **P1** | **NM_203447.3:c.[2606-1G>A; c.405_827del]** | **21** | **F** | 25000 | 0.01 | 9.2 | 24.8 | 2.1 | 15 | 68.7 |
| | P2 | NM_203447.3:c.[2606-1G>A; c.405_827del] | 16 | M | 9140 | 4.17 | 3.72 | 35 | 0.8 | 13 | 67 |
| | P3 | NM_203447.3:c.5625T>G:p.Y1875 | 16 | F | 1690 | 4.2 | 4.63 | 50.3 | 1 | 14 | 59.8 |
| | P4 | NM_203447:c.827+6T>C | 16 | F | 11240 | 5.9 | 4.9 | 11.4 | 0.73 | 15 | 77.4 |
| | P5 | NM_203447.3:c.5962_6068del | 7 | M | 10170 | 5.29 | 9.93 | 26.4 | 0.7 | 17 | 79.5 |
| | P6 | NM_001193536:c.3949+1G>T | 14 | M | 15550 | 6.82 | 4.46 | 11.7 | 1.3 | 15 | 62.5 |
| | P7 | NM_001193536:c.1593+1G>T | 4 | M | 86940 | 5.3 | 24.9 | 31.4 | 9 | 17 | 70.6 |
| | P8 | NM_001193536:c.1593+1G>T | 9 | F | 44630 | 5.26 | 12.49 | 34.8 | 8.1 | 9 | 58.2 |
| | P9 | NM_001190458:c.1905_1905+1delGG | 8 | F | 26340 | 3.81 | 21.42 | 35.1 | 3.2 | 14 | 58.8 |
| | P10 | NM_203447.3:c.5625T>G:p.Y1875 | 21 | M | 265 | 4.7 | 9.7 | 12 | 1.1 | 12 | 70.1 |
| **Average±SEM** | | | **13.2±5.9** | **5/5 (M/F)** | **19817.30±4772.6** | **4.5±0.57** | **10.53±2.3** | **27.29±4.02** | **2.8±0.99** | **14.1±0.75** | **67.26 ±2.37** |
| ATOPIC DERMATITIS | P11 | **DOCK8 mutation negative** | 6 | M | 6540 | 5.2 | 10.6 | 16.5 | 0.9 | 16 | 64.4 |
| | P12 | | 15 | M | 16500 | 6.39 | 3.72 | 15.6 | 1.4 | 14 | 71.3 |
| | P13 | | 10 | M | 1612 | 5.48 | 8.01 | 13.9 | 1.2 | 16 | 73.3 |
| | P14 | | 3 | M | 839 | 5.3 | 6.59 | 6.7 | _ | 13 | 56.1 |
| | P15 | | 8 | M | 1221 | 4.9 | 5.09 | 13 | 1.5 | 9 | 40.8 |
| | P16 | | 13 | M | 659 | 5.2 | 5.15 | 13 | 1.2 | 10 | 54.9 |
| | P17 | | 15 | F | 1387 | 4.71 | 9.63 | 12.3 | 1.4 | 15 | 75 |
| | P18 | | 16 | M | 365 | 5.15 | 2.71 | 7.9 | 2.3 | 14 | 65.1 |
| | P19 | | 12 | M | 4587 | 5.66 | 9.2 | 5.1 | 1.5 | 16 | 70.6 |
| **Average±SEM** | | | **10.8±1.4** | **8/1 (M/F)** | **5288.20±1736.3** | **5.33±0.16** | **6.74±0.92** | **27.29±1.3** | **1.43±0.14** | **13.6±0.86** | **63.5 ± 3.702** |

**Table S2:** Positively identified metabolites that have been significantly changed between the DOCK8 deficient patients and healthy controls. q-value< 0.05

| HMDB ID | Name | Mass (m/z) | Retention time (min) | Fold change | p-value |
|---|---|---|---|---|---|
| 316 | Phenyl-Leucine | 278.1631 | 15.9 | 0.395278 | 0.000060 |
| HMDB00020 | p-Hydroxyphenylacetic acid | 152.0473 | 16.91 | 1.665532 | 0.000897 |
| HMDB00070 | D-Pipecolic acid | 129.079 | 13.23 | 1.587058 | 0.000212 |
| HMDB00112 | Gamma-Aminobutyric acid | 103.0633 | 7.79 | 1.534256 | 0.000000 |
| HMDB00133 | Guanosine | 283.0917 | 2.22 | 4.122263 | 0.000001 |
| HMDB00206 | N6-Acetyl-L-Lysine | 188.1161 | 5.71 | 1.681203 | 0.000000 |
| HMDB00210 | Pantothenic acid | 219.1107 | 8.37 | 1.832101 | 0.000000 |
| HMDB00224 | O-Phosphoethanolamine | 141.0191 | 2.02 | 0.582280 | 0.000076 |
| HMDB00251 | Taurine | 125.0147 | 2.24 | 0.628275 | 0.000000 |
| HMDB00259 | Serotonin | 176.095 | 24.65 | 1.815646 | 0.000003 |
| HMDB00271 | Sarcosine | 89.0477 | 9.34 | 1.573576 | 0.000000 |
| HMDB00300 | Uracil | 112.0273 | 11.34 | 2.245593 | 0.000003 |
| HMDB00440 | 3-Hydroxyphenylacetic acid | 152.0473 | 16.72 | 1.665532 | 0.000897 |
| HMDB00450 | 5-Hydroxylysine | 162.1004 | 13.88 | 2.087091 | 0.000000 |
| HMDB00452 | L-Alpha-aminobutyric acid | 103.0633 | 9.13 | 2.122152 | 0.000000 |
| HMDB00469 | 5-Hydroxymethyluracil | 142.0378 | 8.87 | 0.661088 | 0.000001 |
| HMDB00500 | 4-Hydroxybenzoic acid | 138.0317 | 17.57 | 0.481133 | 0.032552 |
| HMDB00500 | 4-Hydroxybenzoic acid | 138.0317 | 17.57 | 0.497054 | 0.000000 |
| HMDB00669 | Ortho-Hydroxyphenylacetic acid | 152.0473 | 16.42 | 1.665532 | 0.000897 |
| HMDB00684 | L-Kynurenine | 208.0848 | 11.44 | 1.744385 | 0.000000 |
| HMDB00706 | L-Aspartyl-L-phenylalanine | 280.1059 | 10.07 | 0.315026 | 0.000011 |
| HMDB00716 | L-Pipecolic acid | 129.079 | 13.45 | 1.587058 | 0.000212 |
| HMDB00750 | 3-Hydroxymandelic acid | 168.0423 | 21.64 | 1.586043 | 0.000000 |
| HMDB00965 | Hypotaurine | 109.0197 | 2.47 | 0.437627 | 0.000024 |
| HMDB01123 | 2-Aminobenzoic acid | 137.0477 | 16.62 | 3.014851 | 0.000000 |
| HMDB01232 | 4-Nitrophenol | 139.0269 | 23.45 | 1.730548 | 0.000000 |
| HMDB01414 | 1,4-diaminobutane | 88.1 | 21.27 | 2.039329 | 0.000000 |
| HMDB01476 | 3-Hydroxyanthranilic acid | 153.0426 | 18.14 | 1.808715 | 0.000000 |
| HMDB01906 | 2-Aminoisobutyric acid | 103.0633 | 8.91 | 2.122152 | 0.000000 |
| HMDB01918 | Thyroxine | 776.6867 | 27.74 | 1.639578 | 0.000000 |
| HMDB02064 | N-Acetylputrescine | 130.1106 | 7.25 | 2.196515 | 0.000000 |
| HMDB02390 | 3-Cresotinic acid | 152.0473 | 16.8 | 1.665532 | 0.000897 |
| HMDB03012 | Aniline | 93.0578 | 17.32 | 2.035166 | 0.000000 |
| HMDB03334 | Symmetric dimethylarginine | 202.143 | 3.05 | 1.924655 | 0.000000 |
| HMDB03355 | 5-Aminopentanoic acid | 117.079 | 8.68 | 1.622041 | 0.000751 |

| | | | | | |
|---|---|---|---|---|---|
| HMDB03911 | 3-Aminoisobutanoic acid | 103.0633 | 8.67 | 2.122152 | 0.000000 |
| HMDB03911 | 3-Aminoisobutanoic acid | 103.0633 | 16.29 | 1.943611 | 0.000000 |
| HMDB13302 | Phenylalanylphenylalanine | 312.1474 | 16.55 | 0.529397 | 0.000000 |
| HMDB28848 | Glycyl-Phenylalanine | 222.1004 | 11.65 | 0.597854 | 0.000037 |
| HMDB28854 | Glycyl-Valine | 174.1004 | 9.19 | 0.266569 | 0.001304 |

**Table S3.** Positively identified metabolites that have been significantly changed between AD and Control, q-value<0.05.

| HMDB ID | Name | Mass (m/z) | Retention time (min) | Fold change | p-value |
|---|---|---|---|---|---|
| 316 | Phenyl-Leucine | 278.1631 | 15.9 | 0.221546 | 0.000008 |
| HMDB00130 | Homogentisic acid | 168.0423 | 24.84 | 0.512373 | 0.000003 |
| HMDB00133 | Guanosine | 283.0917 | 2.22 | 6.402693 | 0.000000 |
| HMDB00148 | L-Glutamic Acid | 147.0532 | 5.05 | 0.534262 | 0.000000 |
| HMDB00152 | Gentisic acid | 154.0266 | 17.11 | 0.546813 | 0.015066 |
| HMDB00191 | L-Aspartic Acid | 133.0375 | 5.16 | 0.639508 | 0.000498 |
| HMDB00210 | Pantothenic acid | 219.1107 | 8.37 | 1.539231 | 0.000000 |
| HMDB00214 | Ornithine | 132.0899 | 16.58 | 0.579056 | 0.000000 |
| HMDB00224 | O-Phosphoethanolamine | 141.0191 | 2.02 | 0.570539 | 0.000095 |
| HMDB00251 | Taurine | 125.0147 | 2.24 | 0.620118 | 0.000000 |
| HMDB00259 | Serotonin | 176.095 | 24.65 | 2.828641 | 0.000000 |
| HMDB00440 | 3-Hydroxyphenylacetic acid | 152.0473 | 16.72 | 0.660876 | 0.021910 |
| HMDB00450 | 5-Hydroxylysine | 162.1004 | 13.88 | 2.452587 | 0.000000 |
| HMDB00452 | L-Alpha-aminobutyric acid | 103.0633 | 9.13 | 1.775709 | 0.000001 |
| HMDB00500 | 4-Hydroxybenzoic acid | 138.0317 | 17.57 | 0.533774 | 0.035612 |
| HMDB00517 | L-Arginine | 174.1117 | 2.44 | 1.616970 | 0.000000 |
| HMDB00669 | Ortho-Hydroxyphenylacetic acid | 152.0473 | 16.42 | 0.660876 | 0.021910 |
| HMDB00706 | L-Aspartyl-L-phenylalanine | 280.1059 | 10.07 | 0.189421 | 0.000002 |
| HMDB00725 | Trans-4-Hydroxyl-L-Proline | 131.0582 | 5.17 | 1.556155 | 0.000026 |
| HMDB00750 | 3-Hydroxymandelic acid | 168.0423 | 21.64 | 1.571945 | 0.000000 |
| HMDB00991 | 2-aminooctanoic acid | 159.1259 | 19.2 | 2.092571 | 0.000000 |
| HMDB01123 | 2-Aminobenzoic acid | 137.0477 | 16.62 | 1.989600 | 0.000000 |
| HMDB01232 | 4-Nitrophenol | 139.0269 | 23.45 | 1.665664 | 0.000000 |
| HMDB01336 | 3,4-Dihydroxybenzeneacetic acid | 168.0423 | 23.9 | 1.667841 | 0.000002 |
| HMDB01414 | 1,4-diaminobutane | 88.1 | 21.27 | 1.802736 | 0.000000 |
| HMDB01856 | Protocatechuic acid | 154.02661 | 24.51 | 0.560951 | 0.052173 |
| HMDB01906 | 2-Aminoisobutyric acid | 103.0633 | 8.91 | 1.775709 | 0.000001 |
| HMDB01918 | Thyroxine | 776.6867 | 27.74 | 1.624100 | 0.000000 |
| HMDB02064 | N-Acetylputrescine | 130.1106 | 7.25 | 1.798382 | 0.000000 |
| HMDB02390 | 3-Cresotinic acid | 152.0473 | 16.8 | 0.660876 | 0.021910 |
| HMDB03012 | Aniline | 93.0578 | 17.32 | 3.125674 | 0.000953 |
| HMDB03334 | Symmetric dimethylarginine | 202.143 | 3.05 | 1.823252 | 0.000000 |
| HMDB03355 | 5-Aminopentanoic acid | 117.079 | 8.68 | 1.623791 | 0.000000 |
| HMDB03911 | 3-Aminoisobutanoic acid | 103.0633 | 8.67 | 1.775709 | 0.000001 |
| HMDB13243 | Leucyl-phenylalanine | 278.163 | 16.59 | 1.759358 | 0.000002 |
| HMDB13302 | Phenylalanylphenylalanine | 312.1474 | 16.55 | 0.362381 | 0.000000 |
| HMDB28854 | Glycyl-Valine | 174.1004 | 9.19 | 0.096747 | 0.000328 |

**Table S4.** identified metabolites that have been significantly changed between the DOCK8 deficient and AD patients. q-value< 0.05.

| HMDB ID | Name | Mass (m/z) | Retention time (min) | Fold change | p-value |
|---|---|---|---|---|---|
| HMDB00133 | Guanosine | 283.0917 | 2.22 | 1.553199 | 0.006757 |

| | | | | | |
|---|---|---|---|---|---|
| HMDB00191 | L-Aspartic Acid | 133.0375 | 5.16 | 0.552161 | 0.005203 |
| HMDB00965 | Hypotaurine | 109.0197 | 2.47 | 1.898281 | 0.000833 |
| HMDB00991 | 2-aminooctanoic acid | 159.1259 | 19.2 | 1.550839 | 0.005658 |
| HMDB01476 | 3-Hydroxyanthranilic acid | 153.0426 | 18.14 | 0.624233 | 0.000955 |
| HMDB13243 | Leucyl-phenylalanine | 278.163 | 16.59 | 2.415599 | 0.000005 |
| HMDB28848 | Glycyl-Phenylalanine | 222.1004 | 11.65 | 2.091982 | 0.000525 |

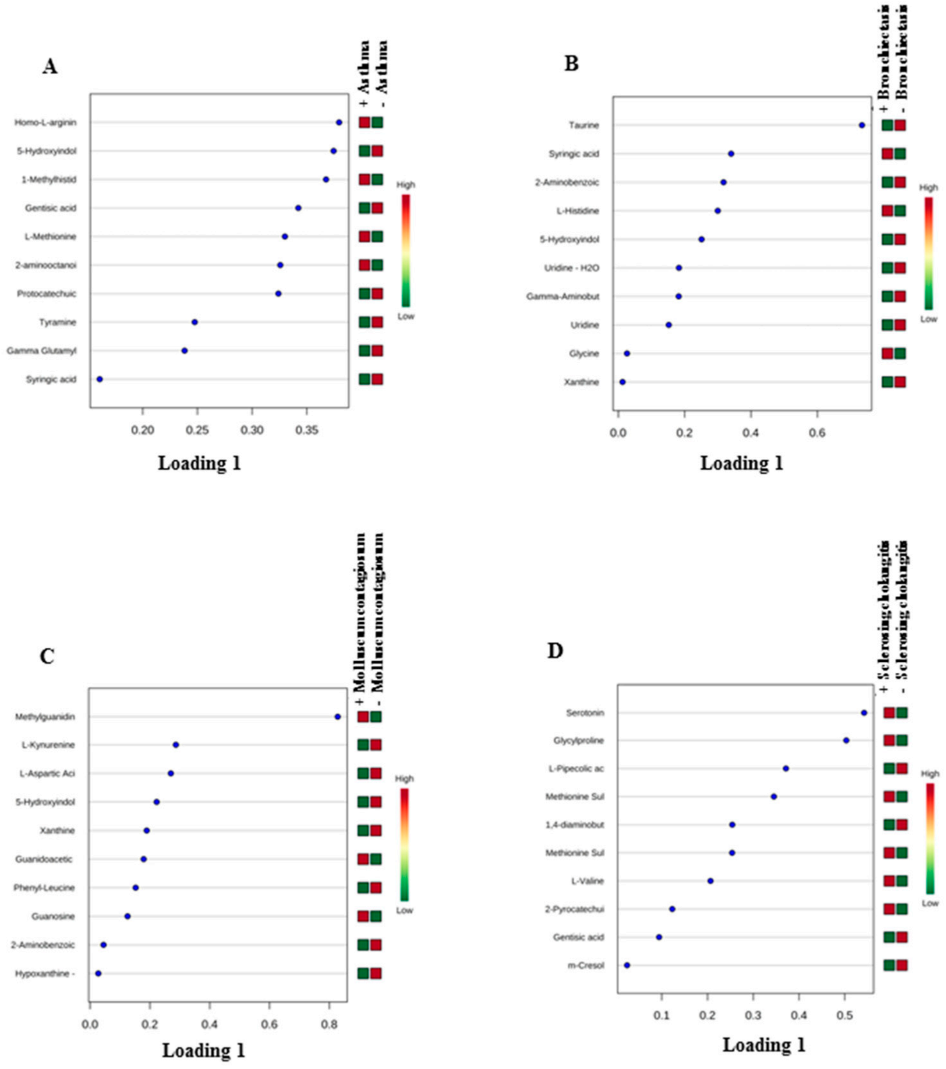


Figure S1A. PLS-DA loading plots based on binary comparisons in DOCK8-deficient patients with (+) and without (-) various clinical phenotypes including (**A**) Asthma, (**B**) Bronchiectasis, (**C**) Molluscum contagiosum, (**D**) Sclerosing cholangitis.

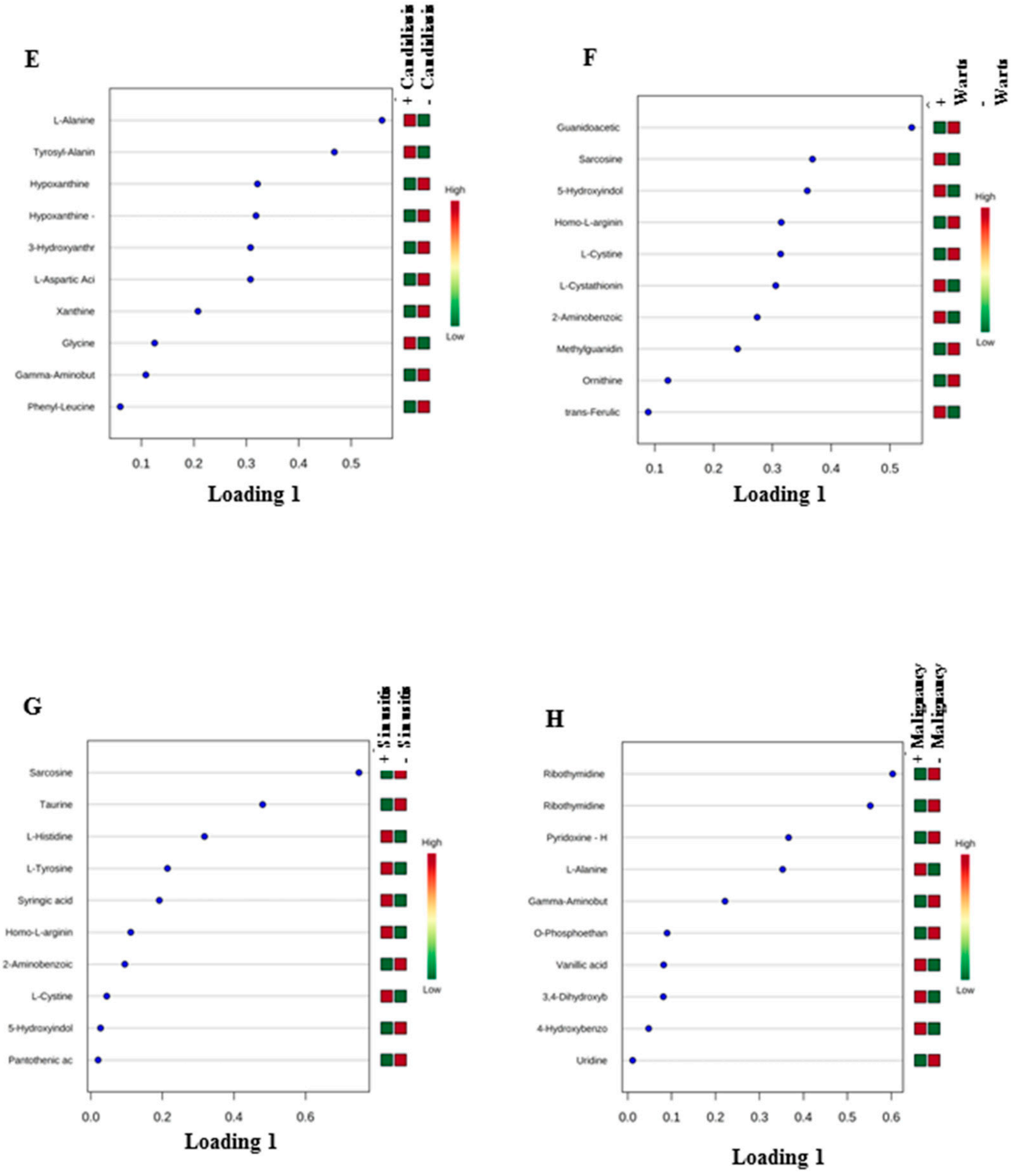


**Figure S1B.** PLS-DA loading plots based on binary comparisons in DOCK8-deficient patients with (**+**) and without (**-**) various clinical phenotypes includin**g** (**E**) Candidiasis, (**F**) Warts, (**G**) Sinusitis, (**H**) Malignancy.
